# Lysine 92 Amino Acid Residue of USP46, a Gene Associated with ‘Behavioral Despair’ in Mice, Influences the Deubiquitinating Enzyme Activity

**DOI:** 10.1371/journal.pone.0026297

**Published:** 2011-10-17

**Authors:** Wei Zhang, Qing-Bao Tian, Qing-Kai Li, Jian-Min Wang, Chao-Nan Wang, Tian Liu, Dian-Wu Liu, Ming-Wei Wang

**Affiliations:** 1 Department of Epidemiology and Statistics, School of Public Health, Hebei Medical University, Shijiazhuang, China; 2 The First Hospital of Hebei Medical University, Brain Aging and Cognitive Neuroscience Laboratory of Hebei Province, Shijiazhuang, China; University of South Florida College of Medicine, United States of America

## Abstract

Deubiquitinating enzymes (DUBs) regulate diverse cellular functions by their activity of cleaving ubiquitin from specific protein substrates. Ubiquitin-Specific Protease 46 (USP46) has recently been identified as a quantitative trait gene responsible for immobility in the tail suspension test and forced swimming test in mice. Mice with a lysine codon (Lys 92) deletion in USP46 exhibited loss of ‘behavioral despair’ under inescapable stresses in addition to abnormalities in circadian behavioral rhythms and the GABAergic system. However, whether this deletion affects enzyme activity is unknown. Here we show that USP46 has deubiquitinating enzyme activity detected by USP cleavage assay using GST-Ub52 as a model substrate. Interestingly, compared to wild type, the Lys 92 deletion mutant resulted in a decreased deubiquitinating enzyme activity of 27.04%. We also determined the relative expression levels of *Usp46* in rat tissues using real-time RT-PCR. *Usp46* mRNA was expressed in various tissues examined including brain, with the highest expression in spleen. In addition, like rat USP46, both human and mouse USP46 are active toward to the model substrate, indicating the USP cleavage assay is a simple method for testing the deubiquitinating enzyme activity of USP46. These results suggest that the Lys 92 deletion of USP46 could influence enzyme activity and thereby provide a molecular clue how the enzyme regulating the pathogenesis of mental illnesses.

## Introduction

Ubiquitin-specific protease (USP), a subfamily of deubiquitinating enzymes (DUBs) [1], [2], is responsible for the removal of ubiquitin or polyubiquitin from target proteins, the processing of ubiquitin precursors, and the disassembly of unanchored polyubiquitin by catalyzing the hydrolysis of isopeptide bonds in ubiquitin-protein conjugates [3]–[7]. USPs have been implicated in wide variety biological processes [8], [9] and involved in the pathogenesis of numerous diseases, including cancer and neurodegeneration [10]–[14]. Recent studies found that USP have also been associated with neurogenetic disorders, including Parkinson's disease [13], [15] and spinocerebellar ataxia [6].

The tail suspension and forced swimming tests [16] are useful experimental paradigms for assessing antidepressant activity and depression-like behavior. In the tests, animals are subjected to inescapable stress of being suspended by their tail or being forced to swim in a water-filled cylinder. The animals rapidly adopt a characteristic immobile posture that has been named ‘behavioral despair’ on the assumption that the animals have given up hope of escaping. Recent study [17] identified USP46 as a quantitative trait gene responsible for the immobility in the tail suspension and forced swimming tests in mice. In this study, mice with a lysine codon (Lys 92) deletion of USP46 showed loss of ‘behavioral despair’ under inescapable stresses in addition to abnormalities in circadian behavioral rhythms and the GABAergic system. The authors demonstrate that USP46 functions to regulate several behavioral processes, including basal immobility, the anti-immobility effects of imipramine, nest building and the muscimol-induced righting reflex. However, comparing the detailed description of the phenotypes of USP46 mutant mice, the molecular mechanism have not been well documented. In particular, whether the lysine codon (Lys 92) deletion in USP46 affects deubiquitinating enzyme activity is unknown.

Here we report that USP46 has deubiquitinating enzyme activity detected by USP cleavage assay using GST-Ub52 as a model substrate. Notably, the Lys 92 deletion mutant results in a decreased deubiquitinating enzyme activity of 27.04% compared to wild type. We also determined the relative expression levels of *Usp46* in rat tissues using real-time RT-PCR. *Usp46* mRNA was expressed in various tissues examined including brain, with the highest expression in spleen. In addition, like rat USP46, both human and mouse USP46 are active toward to the model substrate, suggesting that the USP cleavage assay using GST-Ub52 as a model substrate is simple for testing the deubiquitinating enzyme activity of USP46. These results indicate that the Lys 92 deletion of USP46 could influence deubiquitinating enzyme activity and therefore might contribute to the understanding of neural and genetic mechanisms that underlies the mental disorders associated with USP46.

## Materials and Methods

### Molecular cloning of Usp46

Total RNA was isolated using TRIzol reagent (Tiangen, Beijing, China) from Wistar rat brain (7 weeks old), by the Experimental Animal Center of Hebei Medical University, and human esophageal squamous cell carcinoma cell line TE-1 (Cell Bank of Type Culture Collection of Chinese Academy of Sciences, Shanghai, China), respectively. The cDNA was synthesized by reverse transcription-PCR according to the instructions of Quantscript RT Kit (Tiangen, Beijing, China). Forward primer 5′-GGATCCATGACTGTCCGAAACATCGC-3′ (introducing a *Bam*HIsite) and reverse primer 5′-GTCGACCACGCTCCACCAAGTTATTC-3′ (introducing a *Sal*I site) were used to amplify rat USP46 cDNA. Similarly, human USP46 was amplified using forward primer 5′-GGATCCATGACTGTCCGAAACATCGC-3′ (introducing a *Bam*HIsite) and reverse primer 5′-GTCGACCGCAGGTCTTTCAGTTACTC-3′ (introducing a *Sal*I site). The PCR amplified fragment with a predicted size was subcloned into pGEM-T Easy vector (Promega). *Escherichia coli* DH5α harboring pGEM-*Usp46* clone was cultured to prepare supercoiled recombinant plasmid DNA using a Mini Prep kit (QIAGEN). The DNA sequences were determined by automated sequencing (ABI PRISM).

### Expression GST-USP46 fusion protein in *Escherichia coli* DH5α

#### Plasmid construction

To construct a expression vector suitable for production of glutathione *S*-transferase (GST) fusion protein (GST-USP46) in *Escherichia coli*, we first generated a 1.1-kb DNA fragment containing the coding sequence for rat USP46, and subcloned in-frame with GST into the *Bam*HI and *Sal*Isites of the expression vector pGEX-6p-1 (Amersham Pharmacia Biotech) to generate pGEX-USP46. The recombined plasmid was then used to transform *Escherichia coli* DH5α.

#### Expression in Escherichia coli DH5α

*Escherichia coli* DH5α harboring pGEX-USP46 was incubated in LB medium containing 100 µg of ampicillin per ml at 37°C overnight. The pre culture was diluted 1/10 into fresh LB medium containing 100 µg of ampicillin per ml and incubated at 37°C until an OD 600 of 0.5 is reached, then added IPTG at a final concentration of 1 mM and continued the incubation for 4 h. After the incubation, cells were collected by centrifugation at 15,000× g for 10 min at 4°C, and resuspended in buffer PBS (pH 7.4) containing 140 mM NaCl, 2.7 mM KCl, 10 mM Na_2_HPO_4_, and 1.8 mM KH_2_PO_4_. Cells were disrupted by sonication (six times for 10 s each), then added equal value of 2× SDS sample buffer, boiled for 2 min. The expressed GST-USP46 fusion protein was detected by 10% SDS PAGE.

### USP cleavage assay

The deubiquitinating enzyme activity was detected by USP cleavage assay using GST-Ub52 fusion protein as a model substrate, which has been previously described [18]. The model substrate (GST-Ub52) comprising the natural human Ub52 fusion protein precursor linked to GST, such that the ubiquitin sequences comprise the middle portion of the hybrid protein, was cleaved efficiently by UBP2 (positive control) to yield a product of the expected size. USP46 enzyme activity was assayed basically following the method described by Tian et al. [19]. Briefly, a pAC-T7 plasmid was produced by inserting a T7 promoter from *Bgl*II and *Hind*III fragment of pET-3d (a pBR322 Amp^r^ replicon) into pACYC184 (Cm^r^ replicon) at its *Bam*HI and *Hind*III site. Then, a pAC-T7-*Usp46* plasmid was produced by inserting the complete coding sequence of Usp46 into pAC-T7 plasmid at the *Bam*HI site. The plasmid pACYC-*UBP2* (pACYC184 Cm^r^ replicon) containing yeast UBP2 co-transformed with pGEX-Ub52 as a positive control for USP cleavage assay. For cleavage of the GST-Ub52 substrate, *E. coli* strain BL21 (DE3) cells harboring pGEX-*Ub52* were further transformed with either pAC-T7-*Usp46* or pACYC-*UBP2*, and colonies resistant to both ampicillin and chloramphenicol were grown-up. Protein expression was induced by IPTG and the cells were further incubated for 3 hours, when the USPs cleaved the substrate. Soluble protein extracts were prepared after sonication of the cell lysate. GST fusion proteins and their cleavage products were purified by GSH-Sepharose™ Resin (Sangon Biotech, Shanghai, China) and detected by 10% SDS PAGE. The relative intensity of the resulting bands was analyzed by Odyssey V3.0 software. The arbitrary unit calculated by the ratio of 36 KDa to 45 KDa protein bands was used to measure the relative deubiquitinating enzyme activity of wild type and mutant of USP*46*.

The USP cleavage assay using ubiquitin-β-galactosidase (Ub-Met-β-gal) as a model substrate has been previously described [18]. The substrate is a fusion protein in which the ubiquitin was fused to the N-terminus of β-galactosidase. Upon meeting the active deubiquitinating enzyme in the cell, the substrate was cleaved efficiently and yields a product of the expected size. Plasmid pAC-M-β-gal expresses the Ub-Met-β-gal fusion protein substrate in a pACYC184 Cm^r^ replicon. *E. coli* BL21 (DE3) bacteria harboring pGEX-6P-1-USP46 were transformed with pAC-M-β-gal, Amp^r^ Cm^r^ colonies were grown and induced with IPTG, and total protein extracts were analyzed by Western blotting with anti-β-galactosidase purified monoclonal antibody (Promega) and anti-mouse IgG (H&L) (GOAT) antibody IRDye800CW® Conjugated (Rockland) and detected by the Bio Imaging Navigator FSX100 system (Olympus). The relative intensity of the resulting bands was analyzed by Odyssey V3.0 software.

### Real-time RT-PCR analysis

Real-time RT-PCR analysis (qRT-PCR) was used to measure the expression levels of *Usp46* in Wistar rat tissues (Experimental Animal Center of Hebei Medical University). Reactions utilizing equal amounts of total RNA from each sample were carried out on Rotor Gene 6000 real-time detection system (Bio-Rad) using the Quant one step SYBR Green RT-PCR reagent kit (Tiangen, Beijing, China) according to the instructions. All qRT-PCR were carried out for 40 cycles under the standard PCR conditions (one cycle at 50°C for 30 min, one cycle at 95°C for 2 min and 40 cycles of 94°C for 20 s, 55°C for 20 sec, 68°C for 20 min, and 72°C∼95°C for Melt Curve Analysis). The results were analyzed based on cycle threshold (Ct) values of a method called 2^−ΔΔCt^ method using GAPDH as an internal control. Briefly, a ΔCt was first calculated by subtracting the averaged Ct value of GAPDH from the averaged Ct value of *Usp46* of each tissue, and then a ΔΔCt value was calculated by subtracting the ΔCt values of *Usp46* derived from brain. Fold differences were determined by 2 to the power of minus ΔΔCt. The primers used were: Forward primer 5′-CCGATGTGATTAGCTCATG-3′ and reverse primer 5′- GCCACACACATACAGTAAC-3′.

### Site-directed mutagenesis

The Cys 44 within the *Usp46* conserved Cys-box was mutated to serine and the Lys 92 was deleted using deoxyoligonucleotides and the Stratagene Quik-Change™ site-directed mutagenesis kit according to the manufacturer's instructions using pAC-T7-*Usp46* as a template. The resulting plasmids were pAC-T7-*Usp46* (C44S) and pAC-T7-*Usp46* (ΔK92), respectively. Mutations were confirmed by DNA sequencing.

### Other methods

Animals were handled in accordance with the National Institute of Health *Guide for Care and Use of Laboratory Animals* (NIH Publication no. 80–23). The use of the animals was in agreement with the Animal Care and Use Committee of the Hebei Medical University (Permit Number: 1106110).

## Results

### Molecular cloning of the open reading frame of Usp46 and expression of GST-USP46 fusion protein in *Escherichia coli* DH5α

**Figure 1:**
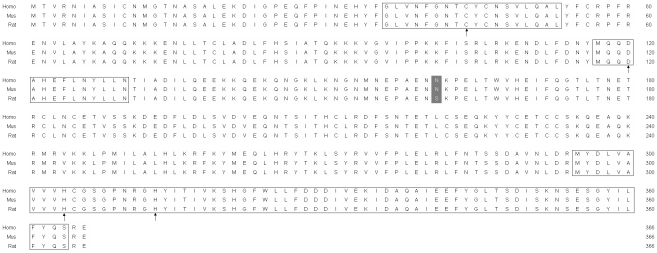
Multi-alignment of amino acid sequences of *Homo sapiens*, *Mus musculus*, and *Rattus norvegicus* of *USP46*. Conserved Cys-, Asp- and His- domains of deubiquitinating enzyme are boxed. The amino acid sequences are highly conserved among the species and different only at the position of 161 (indicated by gray box).

**Figure 2:**
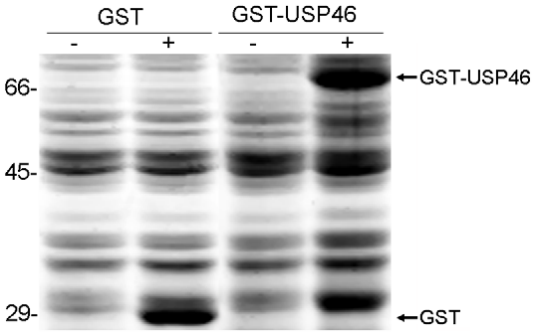
Expression of GST-USP46 fusion protein. GST-USP46 fusion protein was expressed in *Escherichia coli* strain DH5α. Bacteria harboring recombinant plasmid pGEX-*Usp46* were grown up and the expression of GST or GST-USP46 was induced by IPTG (lanes 2 and 4). Attempts to purify the fusion protein were failed, because the protein remained insoluble even after trying a lot of detergents including Triton-100, Urea, and *N*-lauroyl sarcosinate (data not shown).

In order to investigate the deubiquitinating enzyme activity of *Usp46*, we isolated the open reading frame of *Usp46* from rat brain by RT-PCR using a pair of primers designed with reference to sequences of *Rattus norvegicus* (GenBank accession number XM214034). The open reading frame of *Usp46* consists of 1101 bp encoding a polypeptide of 366 amino acid residues, and contains highly conserved Cys, Asp and His domains, which is the characteristic of the ubiquitin-specific proteases [1]. The nucleotide sequence of *Usp46* has been submitted to the GenBank under accession number GU455413. Similarly, we also cloned human *Usp46* from TE-1 cell line (Genbank accession number GU455414). Comparison of the sequence of USP46 of human, rat and mouse revealed 99.7% homology between human and rat and 100% between human and mouse at the amino acid level (Fig. 1). The calculated molecular mass of USP46 is about 42 KDa. As expected, GST-USP46 expressed in the transformants with approximate 68 KDa detected by 10% SDS-PAGE (Fig. 2).

### Deubiquitinating enzyme activity of USP46

**Figure 3:**
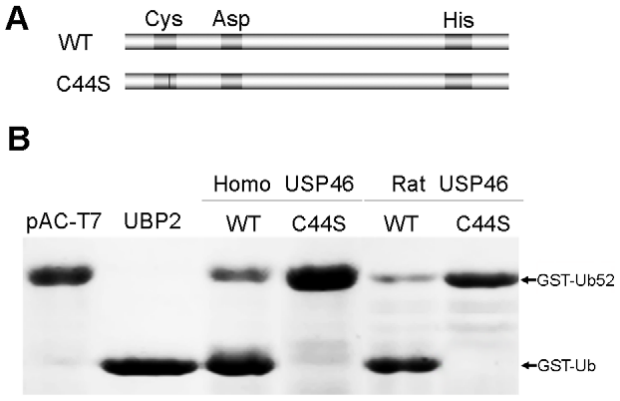
Both human and rat USP46s have deubiquitinating activity toward the model substrate of GST-Ub52. (A) Constructs of wild-type (WT) and mutant USP46 (C44S) for the USP cleavage assay. (B) Cleavage of model substrate GST-Ub52 by USP46. The GST-Ub52 was coexpressed with indicated proteins induced by IPTG in *E coli* BL21. The arrows indicate the GST-Ub52 (about 42 kDa) and its cleavage products GST-Ub (about 36 kDa), which were purified by glutathione-agarose beads and detected by Coomassie Brilliant Blue. UBP2 (a yeast deubiquitinase) and pAC-T7 (an empty plasmid vector) are used as positive and negative controls, respectively.

To investigate whether *Usp46* has deubiquitinating enzyme activity, we performed USP cleavage assay using GST-Ub52 as a model substrate. After induced by IPTG, UBP2 cleaved GST-Ub52 efficiently to yield a product of the expected size of 36 KDa (Fig. 3 B; lane 2). Both human and rat USP46 resulted in the same cleavage product with UBP2, albeit at a reduced efficiency (Fig. 3 B; lane 3, 5). As expected, when Cys 44 was replaced by serine, the activity of USP46 disappeared (Fig. 3 B; lane 4, 6). Thus, USP46 has deubiquitinating enzyme activity.

**Figure 4:**
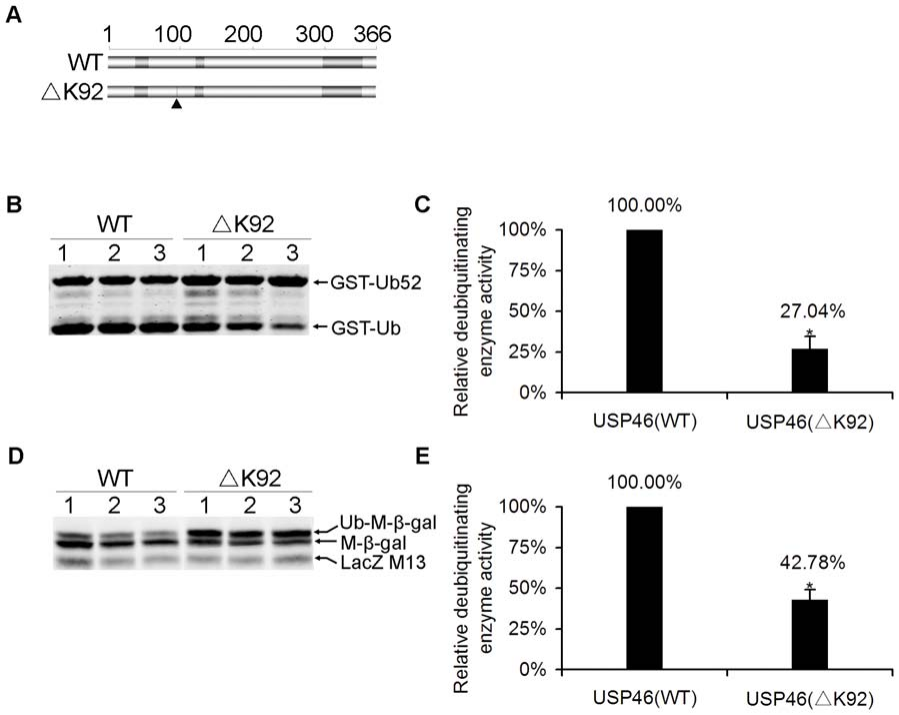
Deubiquitinating enzyme activity of USP46 (ΔK92) declined significantly. (A) Constructs of wild-type (WT) and mutant USP46 (ΔK92) for the USP cleavage assay. The deleted position was indicated by filled triangle. (B) A comparison of the deubiquitinating enzyme activity of wild-type and mutant USP46 (ΔK92) detected by USP cleavage assay using GST-Ub52 as a model substrate (See figure 3 for details). The arrows indicate the GST-Ub52 fusion protein and the cleaved GST-Ub products, respectively. The lane 1, 2, and 3 indicate three independent experiments of the assay. (C) Quantification of enzymatic activity in panel (B). The activity was expressed as the ratio of the intensity of GST-Ub and GST-Ub52 bands. The deubiquitinating enzyme activity of wild-type was defined as 100%. The relative deubiquitinating enzyme activity of ΔK92 mutant was measured compared to the activity of wild-type. Data was shown as mean±SEM of three independent experiments of lane 1, 2, and 3 derived from (B). Values that differ significantly from wild-type (Student's *t* test) are indicated as asterisk (*P*<0.05). (D) A comparison of the deubiquitinating enzyme activity of wild-type and mutant USP46 (ΔK92) detected by USP cleavage assay using Ub-Met-β-gal as a model substrate. The arrows indicate the ubiquitin-β-galactosidase fusion protein (Ub-Met-β-gal), the cleaved β-galactosidase moiety (Met-β-gal) and endogenous β-galactosidase (LacZ-M13) fragments. The lanes 1, 2, and 3 indicate three independent experiments of the assay. (E) Quantification of enzymatic activity in panel (D). The activity was expressed as the ratio of the fluorescence intensity of Met-β-gal and Ub-Met-β-gal bands. The deubiquitinating enzyme activity of wild-type was defined as 100%. The relative deubiquitinating enzyme activity of ΔK92 mutant was measured compared to that of wild-type. Data was shown as mean±SEM of three independent experiments of lane 1, 2, and 3 derived from (D). Values that differ significantly from wild-type (Student's *t* test) are indicated as asterisk (*P*<0.05).

To determine whether Lysine 92 residue affects the deubiquitinating enzyme activity of USP46, we constructed the Lys 92 deletion mutant by site-directed mutagenesis. We employed the sensitive and quantitative Odyssey Infrared Imaging System for Western blot assay based on the intensity of fluorescence of protein band, which can reflect the deubiquitinating enzyme activity. We found that the deubiquitinating enzyme activity of USP46 (ΔK92) was decreased significantly compared with that of wild-type (Fig. 4), detected by USP cleavage assay using both GST-Ub52 and Ub-Met-β-gal as substrates (see Fig. 4 B, D). Results from three independent experiments showed that the relative deubiquitinating enzyme activity decreased to 27.04%±7.50% (mean±SEM) in USP46 (ΔK92) mutant compared with wild-type (*P* = 0.010) using GST-Ub52 as substrate (see Fig. 4 C), while 42.78%±6.47% (mean±SEM; *P* = 0.013) using Ub-Met-β-gal as substrate (see Fig. 4 E). The expression levels of USP46 were no significant differences between wild-type and ΔK92 mutant (see Fig. S1). This result suggests that the Lys 92 deletion influences USP46 deubiquitinating enzyme activity significantly.

### Multi-tissues expression of Usp46

**Figure 5:**
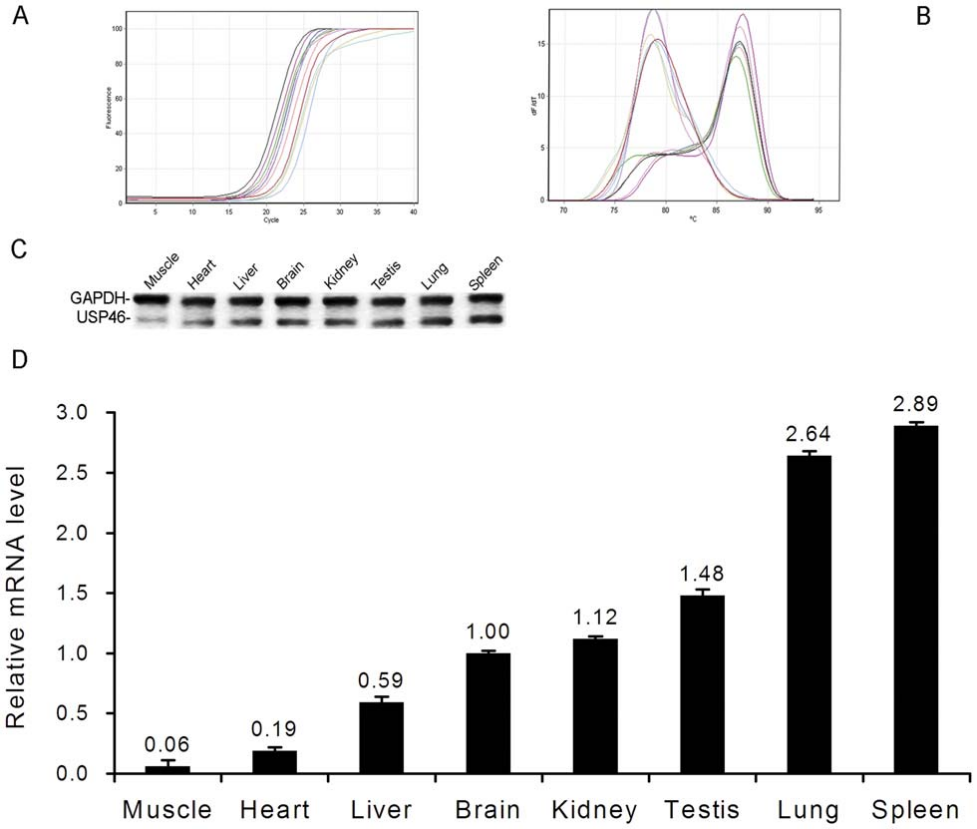
Multi-tissue expression of *Usp46* mRNA. Total RNA was prepared from various tissues and the relative expression levels were analyzed by real- time PCR using GAPDH as an internal control. (A) Fluorescence signals of *Usp46* and *GAPDH* collected by real-time RT-PCR. (B) Melting curves of *Usp46* (left) and *GAPDH* (right) to show the reproducibility of the two genes. (C) Agarose gel analysis of final products of the real-time RT-PCR. The bands of *GAPDH* and *Usp46* were indicated. (D) Bar graph showing the relative levels of *Usp46* mRNA expression in various tissues analyzed by relative quantitative analysis of 2^−ΔΔCt^ method. Note that expression levels were normalized based on the values obtained from the GAPDH gene, comparing with the value of *Usp46* in brain, shown are the means and SEM of the data.

In order to investigate the expression level of *Usp46* in a variety of tissues in rat, real-time RT-PCR analysis using 2^−ΔΔCt^ method was carried out. Approximately, the mRNA of *Usp46* mainly expressed for brain. So we use the expression level of brain as standard, and compared other tissues with brain. The result showed that *Usp46* weakly expressed in skeletal muscle, heart, and liver, which exhibited 0.06, 0.19, and 0.59-fold lower level than brain. While Usp46 expression was observed to be moderate expressed in various organs including kidney and testis (1.12 and 1.48-fold), Usp46 expression was observed to be marginally higher in lung and spleen, which is 2.64 and 2.89-fold higher than brain (Fig. 5). Real-time RT-PCR analysis revealed that the expression of *Usp46* gene is widely distributed in rat tissues, indicating that this enzyme may play a role in these tissues.

## Discussion

In the present study, we report that USP46 has deubiquitinating enzyme activity detected by USP cleavage assay using GST-Ub52 as a model substrate. We find that, compared to wild type, the Lys 92 deletion mutant results in a decreased enzyme activity of 27.04%. This work indicates that behavioral despair associated mutant of the Lys 92 deletion of USP46 could influence enzyme activity and therefore might contribute to the understanding of the molecular mechanisms of the mental disorders associated with USP46. In addition, we also determined the relative expression levels of *Usp46* in rat tissues using real-time RT-PCR. *Usp46* mRNA is expressed in various tissues examined including brain, with the highest expression in spleen.

Our data reveal that USP46 has deubiquitinating enzyme activity detected by USP cleavage assay using GST-Ub52 as a model substrate. Our findings are in good agreement with previous study [20] in the enzyme activity of USP46 detected by the cleavage assay using Ub-Met-β-gal as a substrate using western bolt method. The both methods co-expressed USP and model ubiquitin fusion protein substrate in a bacteria based system, though different model substrate (GST-Ub52 and Ub-Met-β-gal) were used, suggesting that USP46 has deubiquitinating enzyme activity by itself toward the model substrate in bacterial cells.

On the other hand, Cohn MA et al [21] reported that both USP46 and USP12 deubiquitinating enzymes have weak activity by themselves, and that their activities are stimulated by UAF1 strongly. They conclude that the enzyme activity of both USP46 and USP12 are regulated by UAF1. In the study, they assessed the deubiquitinating activity by an *in vitro* deubiquitinating enzyme assay using ubiquitin-AMC [22] as a substrate. The differences in the detection systems might account for the difference in enzyme activity of USP46 between those studies. Cohn et al hired an *in vitro* system using ubiquitin-AMC as a model substrate. The system is relative new, and needs purified enzyme from Sf9 insect cells, since the most USPs is insoluble when expressed in *E. coli*. This is also the case in USP46 (Fig. 2). In contrast, both the current study and Quesada et al [20] used a bacterial expression system using either Ub-Met-β-gal or GST-Ub52 as model substrates. In those systems, the enzyme substrate interaction happens in bacterial cells and no protein purification is needed. In addition, like rat USP46, both human and mouse USP46 are active toward to the model substrate in our detection system (Fig. 3), indicating the USP cleavage assay is a stable method to detect deubiquitinating enzyme activity of USP46.

Usp46 with a Lys 92 deletion is closely associated with loss of ‘behavioral despair’ under inescapable stresses in addition to abnormalities in circadian behavioral rhythms and the GABAergic system [17]. However, the effects of Lys 92 deletion on the deubiquitinating enzyme activity of USP46 were not previously defined. Our results indicate that after deletion of Lys 92, the deubiquitinating enzyme activity of USP46 decreased to 27.04% compared with wild-type by GST-Ub52 assay, while 42.78% by Ub-Met-β-gal assay (Fig. 4). The differences in the detection systems might account for the difference in enzyme activity of USP46 between GST-Ub52 assay and Ub-Met-β-gal assay, but these two assays both revealed that after deletion of Lys 92, the deubiquitinating enzyme activity of USP46 declined significantly, supporting the notion of USP46 as a candidate gene regulating behavioral despairs.

More recently, Itaru Kushima, Branko Aleksic et al. explored an association of USP46 with bipolar disorder and schizophrenia in a Japanese population [23]. They found nominal evidence for an association of rs12646800 and schizophrenia. This association was not significant after correction for multiple testing. No significant association was detected for bipolar disorder. In conclusion, their data argue against the presence of any strong genetic susceptibility factors for bipolar disorder or schizophrenia in the region USP46. However, our finding, that the Lys 92 deletion of USP46 influences enzyme activity, provides a molecular clue in the interpretation how the enzyme regulates the pathogenesis of mental illnesses. In any case, further investigation is clearly needed to determine how the USP46 mutation affects the GABAergic system and involves in mental illnesses.

In conclusion, our data indicate that USP46 in solitary conditions has deubiquitinating enzyme activity detected by USP cleavage assay using GST-Ub52 as a model substrate, which is a simple and stable method to testing the enzymatic activity of USP46. The Lys 92 deletion of USP46 could influence enzyme activity and might contribute to the understanding of the neural and genetic mechanisms that underlie the mental disorders associated with this gene, thereby provide a molecular clue how the enzyme regulating the pathogenesis of mental illnesses.

## Supporting Information

Figure S1 **The expression levels were no significant differences between wild-type and ΔK92 mutant USP46.** *E. coli* strain BL21 (DE3) cells harboring pGEX-*Ub52* were co-transformed with either pAC-T7-*Usp46* (wild-type) or pAC-T7-*Usp46* (ΔK92). Protein expression was induced by IPTG and the cells were further incubated for 3 hours. Total protein extracts were subjected to SDS-PAGE and analyzed by Western blot using anti-T7 rabbit polyclonal antibody. The T7-USP46 was indicated.
(DOC)
