# Supplementary figures and images for: Lysine 92 Amino Acid Residue of USP46, a Gene Associated with ‘Behavioral Despair’ in Mice, Influences the Deubiquitinating Enzyme Activity
Source: PLoS One. 2011 Oct 17;6(10):e26297. doi: 10.1371/journal.pone.0026297 (PMC3197135; doi:10.1371/journal.pone.0026297)

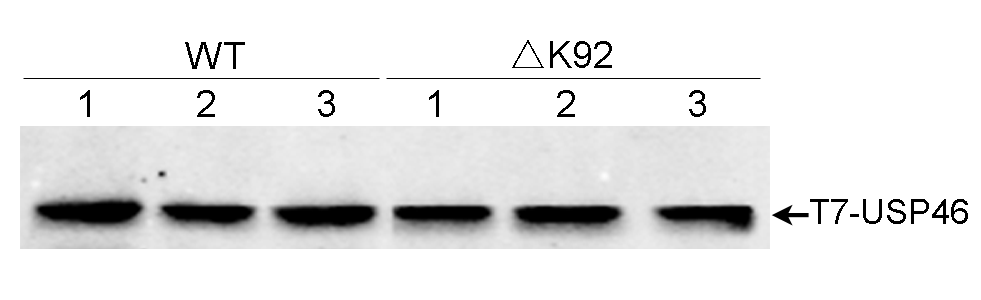

Supplement: Figure S1 — The expression levels were no significant differences between wild-type and ΔK92 mutant USP46. E. coli strain BL21 (DE3) cells harboring pGEX-Ub52 were co-transformed with either pAC-T7-Usp46 (wild-type) or pAC-T7-Usp46 (ΔK92). Protein expression was induced by IPTG and the cells were further incubated for 3 hours. Total protein extracts were subjected to SDS-PAGE and analyzed by Western blot using anti-T7 rabbit polyclonal antibody. The T7-USP46 was indicated. (DOC) [file pone.0026297.s001.doc]
